# Involvement of Palliative Care in Malignant Pleural Mesothelioma Patients and Associations with Survival and End-of-Life Outcomes

**DOI:** 10.3390/curroncol31020076

**Published:** 2024-02-14

**Authors:** Andrew Baird, Abdullah Nasser, Peter Tanuseputro, Colleen Webber, Paul Wheatley-Price, Camille Munro

**Affiliations:** 1 Department of Medicine, University of Ottawa, Ottawa, ON K1H 8L6, Canada; dr.andrew.baird@horizonnb.ca (A.B.); pwheatleyprice@toh.ca (P.W.-P.); 2 Department of Oncology, Western University, London, ON N8W 2X3, Canada; 3 Bruyère Research Institute, Ottawa, ON K1R 6M1, Canada; 4 ICES, University of Ottawa, Ottawa, ON K1Y 4E9, Canada; 5 Ottawa Hospital Research Institute, Ottawa, ON K1Y 4E9, Canada

**Keywords:** palliative care, mesothelioma, survival, emergency departments, patient admission

## Abstract

Malignant pleural mesothelioma is a rare, aggressive, and incurable cancer with a poor prognosis and high symptom burden. For these patients, little is known about the impact of palliative care consultation on outcomes such as mortality, hospital admissions, or emergency department visits. The aim of this study is to determine if referral to supportive and palliative care in patients with malignant pleural mesothelioma is associated with survival and decreased hospital admissions and emergency department visits. This is a retrospective chart review. Study participants include all malignant pleural mesothelioma patients seen at The Ottawa Hospital—an acute care tertiary center—between January 2002 and March 2019. In total, 223 patients were included in the study. The mean age at diagnosis was 72.4 years and 82.5% were male. Of the patients diagnosed between 2002 and 2010, only 11 (9.6%) were referred to palliative care. By comparison, of those diagnosed between 2011 and 2019, 49 (45.4%) were referred to palliative care. Median time from diagnosis to referral was 4.1 months. There was no significant difference in the median survival of patients referred for palliative care compared to those who did not receive palliative care (*p* = 0.46). We found no association between receiving palliative care and the mean number of hospital admissions (1.04 vs. 0.91) from diagnosis to death, and an increase in mean number of emergency department visits in the palliative care group (2.30 vs. 1.18). Although there was increased utilization of palliative care services, more than half of the MPM patients did not receive palliative care despite their limited survival. There was an increase in emergency department visits in the palliative care group; this may represent an increase in the symptom burden (i.e., indication bias) in those referred to palliative care.

## 1. Introduction

Malignant Pleural Mesothelioma (MPM) is a rare, aggressive, and incurable cancer mainly affecting the pleura that is caused by prior exposure to asbestos. MPM prognosis varies by subtype, with epitheliod histology associated with the best prognosis, followed by biphasic and sarcomatoid [1]. However, the overall prognosis is poor, with median overall survival ranging from 8 to 14 months from diagnosis [2]. According to Statistics Canada, about 1.6 of every 100,000 Canadians are diagnosed with mesothelioma annually. In 2017, 490 Canadians died from mesothelioma, while it is estimated that globally, 38,400 people die from the disease each year [3,4]. Although prognosis has improved with palliative care in recent years, systemic medical treatment (e.g., single-agent chemotherapy, multi-agent chemotherapy), surgery, and improved diagnostic methods, patients with MPM have a high burden of physical and psychological symptoms [5,6].

The World Health Organization defines palliative care as “an approach that improves the quality of life of patients and their families facing the problem associated with life-threatening illness, through the prevention and relief of suffering by means of early identification and impeccable assessment and treatment of pain and other problems, physical, psychosocial and spiritual” [7]. Studies have shown, for example, that cancer patients who had early referral to palliative care had less aggressive treatments and improved survival, symptom burden, and quality of life [8,9,10]. Unfortunately, the symptom burden in patients with MPM at the end of life is significant and, due to factors such as delayed diagnosis, palliative care is often introduced late in the disease course [11]. MPM can cause a myriad of symptoms, the more common of which include dyspnea, chest wall pain, anorexia, fatigue, and weight loss. However, there is limited evidence of the involvement and impact of early palliative care on MPM. The RESPECT-MESO multi-center, randomized trial demonstrated that in patients with newly diagnosed MPM with higher performance status, early referral to specialist palliative care did not grant any benefit in quality of life and/or mood [12]. There have been no studies, to our knowledge, that have evaluated the involvement of palliative care on survival and healthcare use by those with MPM.

The objective of this study was to evaluate the associations between referral to palliative care for patients with MPM and their survival and healthcare utilization, including hospital admissions and emergency department visits.

## 2. Materials and Methods

We conducted a retrospective chart review of all individuals diagnosed with MPM at The Ottawa Hospital and followed by The Ottawa Hospital between January 2002 and March 2019. This cohort was part of a longer-term analysis of mesothelioma patients dating back to 1991 and published elsewhere [13]. The Ottawa Hospital is a 1202-bed tertiary care teaching hospital comprising three campuses located in Ottawa, Ontario, Canada. The cancer center is the sole provider of oncology services in the region, and as such receives all the MPM referrals. Across the 17-year study period, The Ottawa Hospital saw an average of 13 MPM patients each year. The Supportive and Palliative Care team at The Ottawa Hospital consists of a consulting service of palliative medicine physicians, nurses who specialize in palliative care, and social workers. We captured those with MPM who received specialist palliative care and/or were known from their Ottawa Hospital charts to be referred to a palliative care physician in the community.

Adult patients aged ≥ 18 with MPM were included in the study. Those with non-pleural mesothelioma were excluded. The primary outcome was overall survival, defined as the time from diagnosis to time of death or last follow-up. Secondary outcomes were emergency department visits and hospital admissions between the date of mesothelioma diagnosis and death or the study cut-off date. To evaluate changes in standards of care over time, referral patterns were compared between 2002–2010 and 2011–2019. An independent reviewer (A.B.) extracted the following information from patients’ paper charts and electronic medical records: baseline demographics, diagnoses, reason(s) for specialist palliative care referral, involvement of palliative care, hospital admissions, and emergency department visits. Patients were typically seen every 1–3 months until death and data on death date were almost fully complete. For a small number with unclear death status, we queried other regional hospital databases and examined published obituaries.

Statistical analyses were carried out in R software v4.0.3 (R Foundation for Statistical Computing, Vienna, Austria). Descriptive statistics were used to estimate the frequencies, means, and standard deviations of the study variables, including age, sex, treatment modalities, and survival. The Kaplan–Meier method was used to obtain estimates of median survival by palliative care involvement (yes/no), with corresponding two-sided 95% confidence intervals. Differences between survival curves were assessed using the log-rank test. Cox proportional hazard models with complete case analysis were used to calculate the hazard ratio for the palliative and non-palliative care groups, controlling for sex, age, performance status, histology, stage, and treatment modalities received (chemotherapy, radiation therapy, and extrapleural pneumonectomy). Multivariate linear regression analyses were performed to compare the number of emergency visits and hospital admissions between the palliative care and non-palliative care groups, controlling for demographic and treatment factors. A *p*-value of 0.05 was used to determine statistical significance. This study was approved by the Ottawa Health Sciences Network Research Ethics Board (20190034-01H).

## 3. Results

All 223 patients with MPM were included in the study. The mean age at diagnosis was 72.4 years, and the percentage of male patients was 82.5%. A majority of patients were previous or current smokers (63.2%), had known asbestos exposure (57.0%), and were diagnosed with epithelioid histology (56.5%). A total of 60 (36.8%) patients had been referred to the specialist palliative care team. Of the 115 patients diagnosed between 2002 and 2010, 11 (9.6%) were referred to the specialist palliative care team either in hospital or in the community. By comparison, of 108 patients diagnosed between 2011 and 2019, 49 (45.4%) were referred to the specialist palliative care team. The median time from diagnosis to referral was 4.12 months and the median time from referral to death was 1.65 months. Female patients were more likely to be referred to palliative care than male patients (25.0% versus 14.7%) but were otherwise similar in baseline demographic and disease characteristics (Table 1).

**Figure 1:**
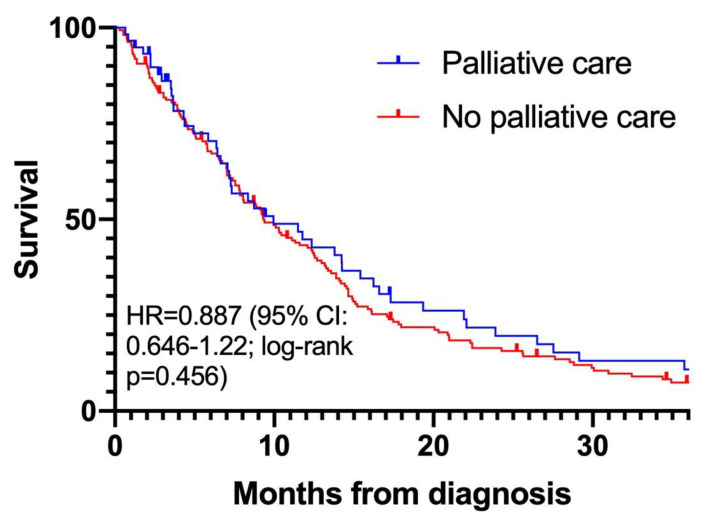
Survival of malignant pleural mesothelioma patients referred to palliative care (blue) compared to those not referred to palliative care (red).

At the end of the study follow-up, 200 (89.7%) patients were deceased. Median overall survival was 9.4 (8.1–12.3) months from diagnosis. There was no statistically significant difference in the median overall survival from diagnosis between patients who received palliative care and those who did not receive palliative care: 10.0 (7.2–16.2) months compared to 9.3 (7.9–12.4) months, respectively (Figure 1; *p* = 0.46; Table S1). The mean numbers of hospital admissions from the time of diagnosis to death or study end-date for the palliative and non-palliative groups were similar: 0.91 and 1.0, respectively. Conversely, an increased number of emergency department visits was seen in the palliative care group (mean 2.30 vs. 1.21).

Several variables in our Cox proportional hazards model were related to a higher risk of death, including a higher Eastern Cooperative Oncology Group (ECOG) performance status scale (i.e., greater disability), non-epitheliod subtypes in histology, and more advanced stage (Table 2). After controlling for these variables and additional variables, including age, sex, chemotherapy, extrapleural pneumonectomy, and radiation therapy, receiving palliative care was not associated with significant differences in survival (HR = 0.77; 95% C.I., 0.53–1.13; *p* = 0.2; Table 2). Controlling for the same factors, palliative care was an independent predictor of increased emergency department visits (β = 1.16; SE = 0.46; *p* = 0.013), but not hospital admissions (β = 0.38; SE = 0.232; *p* = 0.11; Table S2 and Table S3).

## 4. Discussion

Palliative care utilization among those with mesothelioma increased over time but remained low overall at 39.8%. This may, however, be an underestimate, as our data focused on capturing patients referred to the palliative care group in our cancer center and those referred to the community palliative care services in our region but did not capture patients managed by their own family physicians or those who were referred to palliative care after discharge from The Ottawa Hospital program (e.g., to the regional palliative care team). The perceived delay in referral to palliative care in our center may also be skewed by the local practice of our oncology group, as they often provide simultaneous care of symptom management and systemic therapy early on. Female patients were more likely to be referred to palliative care, which is consistent with cancer palliative care trends and patient preferences [14]. Nevertheless, the percentage of those receiving palliative care and the timing of receipt is close to what is observed in the general population of all decedents in Ontario [15].

The data highlighted here provides a real-world analysis of the challenges and poor prognosis associated with mesothelioma. The disease often presents at advanced stages and patients are rarely surgical candidates (only 4 of 122 patients diagnosed between 2010 and 2019 received extrapleural pneumonectomy). Despite new data emerging over the last decade demonstrating a small but significant improvement in overall survival with combination therapy (i.e., Pemetrexed and Cisplatin) [16], patients in our center have had no significant improvement in median survival over the last three decades [13]. Similarly, in our robust and long-term follow-up of these patients, and despite an increasing number of referrals to specialist palliative care, we saw no association between specialist palliative care involvement and overall survival. Ultimately, this is not unexpected, as the median survival from diagnosis to death remained low, at 8.8 months. Furthermore, the median time from specialist palliative care referral to death was short, at 1.65 months, indicating referrals were provided late in the course of the disease. Delayed diagnoses may contribute to these late introductions to palliative care [11]. Patients referred to specialist palliative care also had poorer performance status and were often referred after completion of chemotherapy, which may explain the limited impact and increase in ED visits in this population. The increase in ED visits may also be related to some specialist palliative care involvement arising following an ED visit (i.e., involvement of in-hospital specialist palliative care for those admitted and/or connection to community specialist palliative care following discharge).

Over the last three decades, the management of mesothelioma and malignancy has continued to steadily change and expand [17]. In patients with MPM, this has included new standards in systemic therapy and a smaller focus on surgical intervention. During this timeframe, early involvement of palliative care—especially in disease states with high physical and psychological symptom burden—has undergone more focused investigation. Early palliative care involvement, for example, has been shown to improve quality of life, mood, transition to hospice services, cancer treatment close to death, pulmonary function, and survival in patients with metastatic non-small cell lung cancer [6,18,19,20,21]. During the last 20 years, access to palliative care has significantly improved at the study hospital site. In 1998, only two outpatient clinics were run per week. In 2005, this increased to three clinics per week and since 2019, seven clinics per week are available at the cancer center where MPM patients are treated. There has also been improved access to palliative care in the community. This provided a unique opportunity for our study to assess whether improved access to care would lead to improved utilization and patient outcomes.

This study has several limitations, including its small sample size and retrospective nature. This study did not measure patient-oriented outcomes such as psychosocial well-being or pain and symptom management. The chart review also did not allow an in-depth exploration of reasons for requesting specialist palliative care, and therefore, we do not have reliable longitudinal data measuring the quality of life. Additionally, the Ottawa Hospital charts may not contain a complete record of what regional palliative care resources, including family physicians, may have been utilized in patients’ care, and the lack of documented prior palliative care involvement may offer an inaccurate representation. The levels of involvement in palliative care received were not measured, and there is likely heterogeneity in palliative care between patients. Similarly, we did not capture long-term care, home care, or informal care that may impact acute healthcare use. There was likely indication bias for those who were referred to palliative care as they often had poorer performance status. Finally, this study is from a single academic acute tertiary health center, and therefore its unique institutional practice patterns may limit generalizability. Future research should examine how palliative care in patients with MPM impacts the quality of life outcomes such as pain, symptom burden, or place of death.

## 5. Conclusions

This retrospective chart review is unique in that we have been able to follow patient experiences over a prolonged period at a single center. During the timeframe of this review, from 2002 to 2019, the standard care of patients with mesothelioma has changed; however, performance status and prognosis remain extremely poor. This is a unique scenario in which palliative care, as defined above, should be a crucial part of the management plan in patients with MPM. Similar to previous studies, palliative care involvement was not associated with a decline in survival. The frequency of referral for this service in our center has improved; we hope this trend continues and hope to see earlier integration of palliative care in the care of patients with malignant pleural mesothelioma.

## Figures and Tables

**Table 1:** Baseline characteristics of malignant pleural mesothelioma patients.

	Non-Palliative	Palliative	Total
	(*n =* 163)	(*n =* 60)	(*n =* 223)
Age at diagnosis			
Mean (SD)	72.4 (9.49)	72.3 (10.1)	72.4 (9.63)
Sex			
Female	24 (14.7%)	15 (25.0%)	39 (17.5%)
Male	139 (85.3%)	45 (75.0%)	184 (82.5%)
Smoking status			
Non-smoker	60 (36.8%)	17 (28.3%)	77 (34.5%)
Smoker	100 (61.3%)	41 (68.3%)	141 (63.2%)
Unknown	3 (1.8%)	2 (3.3%)	5 (2.2%)
Asbestos exposure			
No exposure	69 (42.3%)	20 (33.3%)	89 (39.9%)
Exposure	91 (55.8%)	36 (60.0%)	127 (57.0%)
Unknown	3 (1.8%)	4 (6.7%)	7 (3.1%)
ECOG Performance Status			
0 (Fully active)	30 (18.4%)	7 (11.7%)	37 (16.6%)
1	67 (41.1%)	25 (41.7%)	92 (41.3%)
2	34 (20.9%)	12 (20.0%)	46 (20.6%)
3	15 (9.2%)	10 (16.7%)	25 (11.2%)
4 (Fully disabled)	4 (2.5%)	1 (1.7%)	5 (2.2%)
Unknown	13 (8.0%)	5 (8.3%)	18 (8.1%)
IMIG stage			
1	48 (29.4%)	24 (40.0%)	72 (32.3%)
2	18 (11.0%)	4 (6.7%)	22 (9.9%)
3	44 (27.0%)	16 (26.7%)	60 (26.9%)
4	48 (29.4%)	15 (25.0%)	63 (28.3%)
Unknown	5 (3.1%)	1 (1.7%)	6 (2.7%)
Histology			
Biphasic	19 (11.7%)	9 (15.0%)	28 (12.6%)
Epithelial	93 (57.1%)	33 (55.0%)	126 (56.5%)
Other/unknown	29 (17.8%)	7 (11.7%)	36 (16.1%)
Sarcomatoid	22 (13.5%)	11 (18.3%)	33 (14.8%)
EPP			
No EPP	150 (92.0%)	57 (95.0%)	207 (92.8%)
EPP	13 (8.0%)	3 (5.0%)	16 (7.2%)
Radiation therapy			
No radiation	93 (57.1%)	35 (58.3%)	128 (57.4%)
Radiation	68 (41.7%)	24 (40.0%)	92 (41.3%)
Unknown	2 (1.2%)	1 (1.7%)	3 (1.3%)
Chemotherapy			
No chemotherapy	68 (41.7%)	24 (40.0%)	92 (41.3%)
Chemotherapy	95 (58.3%)	36 (60.0%)	131 (58.7%)

ECOG: Eastern Cooperative Oncology Group; IMIG: International Mesothelioma Interest Group; EPP: extrapleural pneumonectomy.

**Table 2:** Cox proportional hazards model for survival of patients with mesothelioma.

Characteristics	HR ^1^	95% CI ^1^	*p*-Value
Palliative care	0.77	0.53, 1.13	0.2
Male sex	1.34	0.89, 2.03	0.2
Age at diagnosis	1.01	0.99, 1.03	0.3
Chemotherapy	0.51	0.34, 0.78	0.002
EPP ^1^	0.9	0.45, 1.80	0.8
Radiation therapy	0.7	0.50, 0.98	0.039
ECOG ^1^			
0	Reference		
1	1.31	0.84, 2.03	0.2
2	1.97	1.16, 3.35	0.012
3	1.45	0.75, 2.81	0.3
4	12.9	4.43, 37.6	<0.001
Histology			
Epithelial	Reference		
Biphasic	1.95	1.17, 3.24	0.01
Sarcomatoid	1.86	1.15, 3.03	0.012
Other/unknown	1.49	0.95, 2.34	0.082
IMIG ^1^ Stage			
1	Reference		
2	1.09	0.62, 1.91	0.8
3	1.54	1.00, 2.36	0.048
4	2.23	1.45, 3.43	<0.001

^1^ HR = Hazard Ratio, CI = Confidence Interval, EPP = extrapleural pneumonectomy, ECOG = Eastern Cooperative Oncology Group, IMIG = International Mesothelioma Interest Group.

## Supplementary Materials

The following supporting information can be downloaded at: https://www.mdpi.com/article/10.3390/curroncol31020076/s1, Table S1: Number of hospital admissions and Emergency Room (ER) visits, by palliative care status; Table S2: Regression analysis of Emergency Visits; Table S3: Regression analysis of hospital visits.
