# Supplementary material for: Involvement of Palliative Care in Malignant Pleural Mesothelioma Patients and Associations with Survival and End-of-Life Outcomes
Source: Curr Oncol. 2024 Feb 14;31(2):1028–34. doi: 10.3390/curroncol31020076 (PMC10888381; doi:10.3390/curroncol31020076)
Supplement: Supplementary file 1 [file curroncol-31-00076-s001.zip › curroncol-2645669-supplementary.pdf]

Supplementary data

Table S1: Number of hospital admissions and Emergency Room (ER) visits, by palliative care status.

	**Non-palliative group**	**Palliative group**
**Hospital admissions**	% patients (n)	% patients (n)
0	50.5 (49)	40.4 (21)
1	25.8 (25)	32.7 (17)
2	12.4 (12)	21.2 (11)
3	5.2 (5)	3.8 (2)
4+	6.2 (6)	1.9 (1)
**ER visits**		
0	47.2 (34)	21.2 (7)
1	27.8 (20)	21.2 (7)
2	9.7 (7)	21.2 (7)
3	2.8 (2)	15.2 (5)
4+	12.5 (9)	21.2 (7)

Table S2: Regression analysis of Emergency Visits

Predictors	*Estimates*	*CI*	*p*
(Intercept)	3.04	-1.38 – 7.45	0.175
Palliative care	1.16	0.25 – 2.06	0.013
Age at diagnosis	-0.01	-0.07 – 0.04	0.626
Male sex	-0.17	-1.33 – 1.00	0.777
Smoker	-0.38	-1.30 – 0.53	0.405
Asbestos Exposure	-0.4	-1.31 – 0.50	0.377
ECOG 1	-0.36	-1.50 – 0.77	0.523
ECOG 2	-0.64	-2.03 – 0.74	0.359
ECOG 3	-0.05	-1.86 – 1.76	0.957
ECOG 4	-2.54	-6.83 – 1.75	0.242
Stage II	0.75	-0.71 – 2.21	0.311
Stage III	0.03	-1.02 – 1.08	0.955
Stage IV	-0.09	-1.23 – 1.06	0.881
Biphasic histology	-1.18	-2.52 – 0.15	0.081
Unknown/other histology	-0.08	-1.22 – 1.06	0.886
Sarcomatoid histology	-0.08	-1.30 – 1.13	0.893
EPP	0.14	-1.37 – 1.64	0.856
Radiation therapy	0.31	-0.57 – 1.19	0.482
Chemotherapy	-0.04	-1.07 – 0.99	0.933
R^2 / R^2 adjusted	0.209 / 0.019		

ECOG: Eastern Cooperative Oncology Group; EPP = extrapleural pneumonectomy

Table S3: Regression analysis of hospital visits

Predictors	*Estimates*	*CI*	*p*
(Intercept)	1.19	-0.94 – 3.32	0.272
Palliative care	0.38	-0.08 – 0.83	0.108
Age at diagnosis	0	-0.03 – 0.02	0.774
Male sex	-0.13	-0.69 – 0.44	0.659
Smoker	-0.3	-0.76 – 0.17	0.214
Asbestos Exposure	-0.24	-0.69 – 0.21	0.294
ECOG 1	0.01	-0.58 – 0.61	0.969
ECOG 2	0.34	-0.36 – 1.05	0.337
ECOG 3	0.11	-0.79 – 1.02	0.806
ECOG 4	-0.19	-1.53 – 1.14	0.777
Stage II	0.24	-0.52 – 1.01	0.525
Stage III	0.11	-0.44 – 0.67	0.683
Stage IV	-0.23	-0.82 – 0.37	0.454
Biphasic histology	-0.45	-1.17 – 0.26	0.208
Unknown/other histology	0.04	-0.57 – 0.64	0.909
Sarcomatoid histology	-0.2	-0.80 – 0.39	0.494
EPP	1.78	0.96 – 2.60	<0.001
Radiation therapy	0.49	0.04 – 0.95	0.035
Chemotherapy	-0.03	-0.54 – 0.48	0.9
R^2 / R^2 adjusted	0.317 / 0.204		

ECOG: Eastern Cooperative Oncology Group; EPP = extra-pleural pneumonectomy
